# Spatial data analysis for intelligent buildings: Awareness of context and data uncertainty

**DOI:** 10.3389/fdata.2022.1049198

**Published:** 2022-11-07

**Authors:** Huan Li, Tiantian Liu, Harry Kai-Ho Chan, Hua Lu

**Affiliations:** ^1^Department of Computer Science, Aalborg University, Aalborg, Denmark; ^2^Department of People and Technology, Roskilde University, Roskilde, Denmark; ^3^Information School, University of Sheffield, Sheffield, United Kingdom

**Keywords:** spatial data uncertainty, context-aware computing, indoor spaces, smart buildings, mobility analysis, IoT data quality

## Abstract

Intelligent buildings are among the most active Internet-of-Things (IoT) verticals, encompassing various IoT-enabled devices and sensing technologies for digital transformation. Analysis of spatial data, a very common type of data collected in intelligent buildings, offers a lot of insights for many purposes such as facilitating space management and enhancing the utilization efficiency of buildings. In this paper, we recognize two major challenges in spatial data analysis for intelligent buildings (SDAIB): (1) the complicated analytical contexts that are related to the building space and internal entities and (2) the uncertainty of spatial data due to the limitations of positioning and other sensing technologies. To address these challenges, we identify and categorize different kinds of analytical contexts and spatial data uncertainties in SDAIB, and propose a unified modeling framework for handling them. Furthermore, we showcase how the proposed framework and the associated modeling techniques are used to enable context-aware and uncertainty-aware SDAIB, in the tasks of hotspot discovery, path planning, semantic trajectory generation, and distance monitoring. Finally, we offer several research directions of SDAIB, in line with the emerging trends of the IoT.

## 1. Introduction

Driven by the popularity of the Internet of Things (IoT) infrastructure, the digital transformation in the architecture industry is experiencing rapid growth. As a well-recognized notation, *intelligent buildings* (or smart buildings) (Qolomany et al., 2019; Daissaoui et al., 2020) formulate a paradigm that integrates various IoT-enabled devices, sensing technologies, and big data analysis technologies for achieving improved efficiency of building uses and enhanced comfort of residents. According to a report^1^ by the research firm MarketsandMarkets, the global market of intelligent buildings is expected to grow from 66.3 billion in 2020 to 108.9 billion in 2025, at a compound annual growth rate of 10.5%.

**Figure 1:**
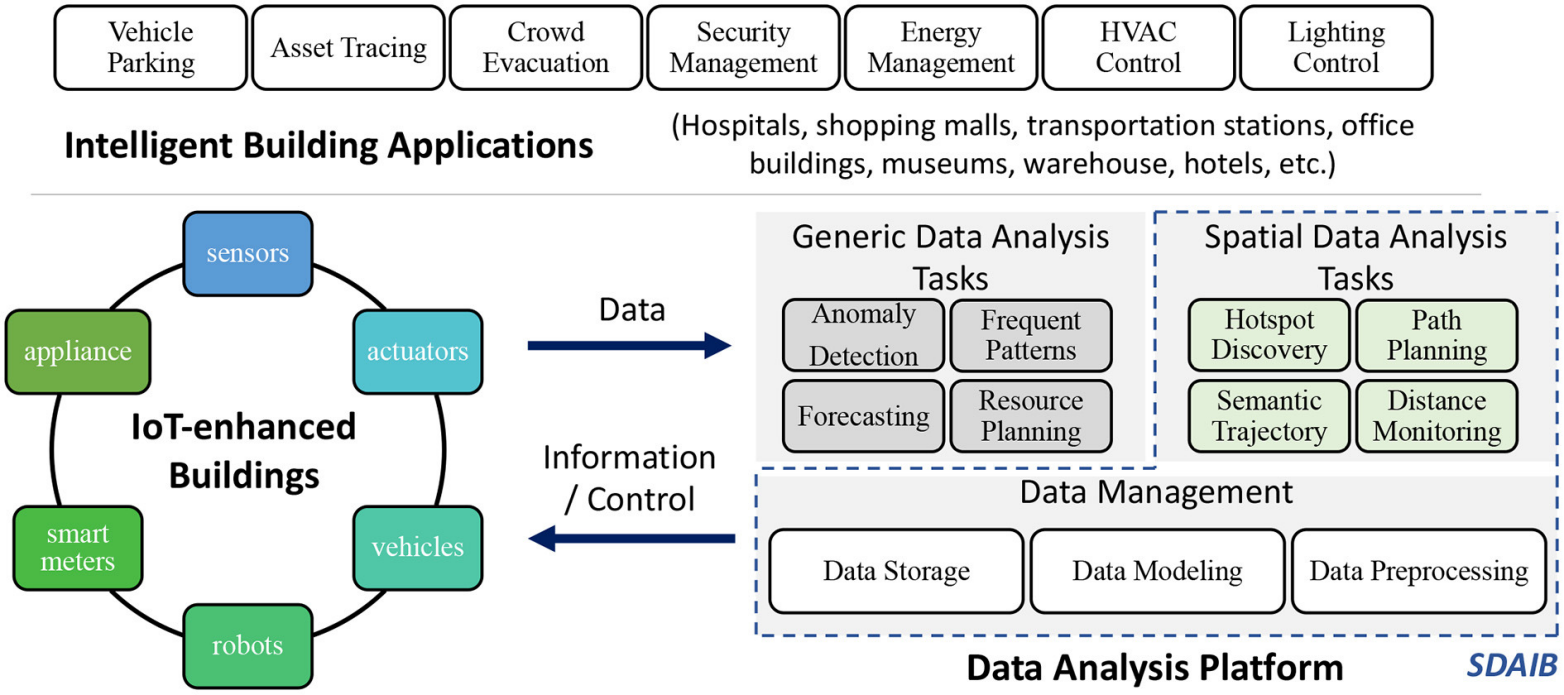
An overall picture of intelligent buildings: IoT devices, data analysis, and applications.

Intelligent buildings are thought of as an ecosystem made up of both hardware and software (Daissaoui et al., 2020). Based on this, Figure 1 presents an overall picture of intelligent buildings, which includes three key components, namely the IoT-enhanced buildings (lower left corner), the data analysis platform (lower right corner), and the intelligent building applications (upper side). In particular, the *IoT-enhanced buildings* encapsulate a wide variety of IoT-enabled devices such as sensors, actuators, and robots that bridge the physical building space and the cyber space. As the underlying infrastructure, the IoT-enhanced buildings employ various sensing technologies to collect data, which are then sent to the data analysis platform. The *data analysis platform* plays the central role in the ecosystem, extracting useful information and generating control messages from the collected data, which in turn facilitate the sensing and interactions within the IoT-enhanced buildings. In the data analysis platform, the fundamental data management module is responsible for data preprocessing, modeling, and storage, on which various data analysis tasks are built. Finally, on top of the IoT-enhanced buildings and the data analysis platform, *intelligent building applications* are designed and tailored for many different building types like hospitals, office buildings, and transportation stations. Some typical applications are asset tracing (Jensen et al., 2009a; Oztekin et al., 2010; Kim et al., 2014), HVAC (heating, ventilation, and air conditioning) control (Capozzoli et al., 2017), and crowd evacuation (Chen and Feng, 2009; Kamkarian and Hexmoor, 2012; Li et al., 2018b).

The management of an intelligent building is often required to be aware of the statuses of many key places of the building. As a result, the data collected in intelligent buildings are often associated with spatial attributes. For example, a smart meter record contains the ID of the room that consumes energy (Ni et al., 2016), while indoor positioning data usually captures a target object's whereabouts as a specific two- or three-dimensional geometric coordinate in the building (Liu et al., 2007; Li et al., 2020b). Correspondingly, a good number of fundamental data analyses for intelligent buildings are oriented to such spatial data, making efforts on facilitating space management and enhancing the utilization efficiency of the building. In this study, we pay particular attention to such *spatial data analysis for intelligent buildings* (SDAIB). Referring to the data analysis platform in Figure 1, general data analysis tasks include anomaly detection, frequent pattern mining, and forecasting, while typical SDAIB tasks are hotspot discovery (Li et al., 2018b,c), path planning (Feng et al., 2020; Chan et al., 2021; Liu et al., 2021a,c), semantic trajectory generation (Li et al., 2020a,b), and distance monitoring (Chan et al., 2022).

**Figure 2:**
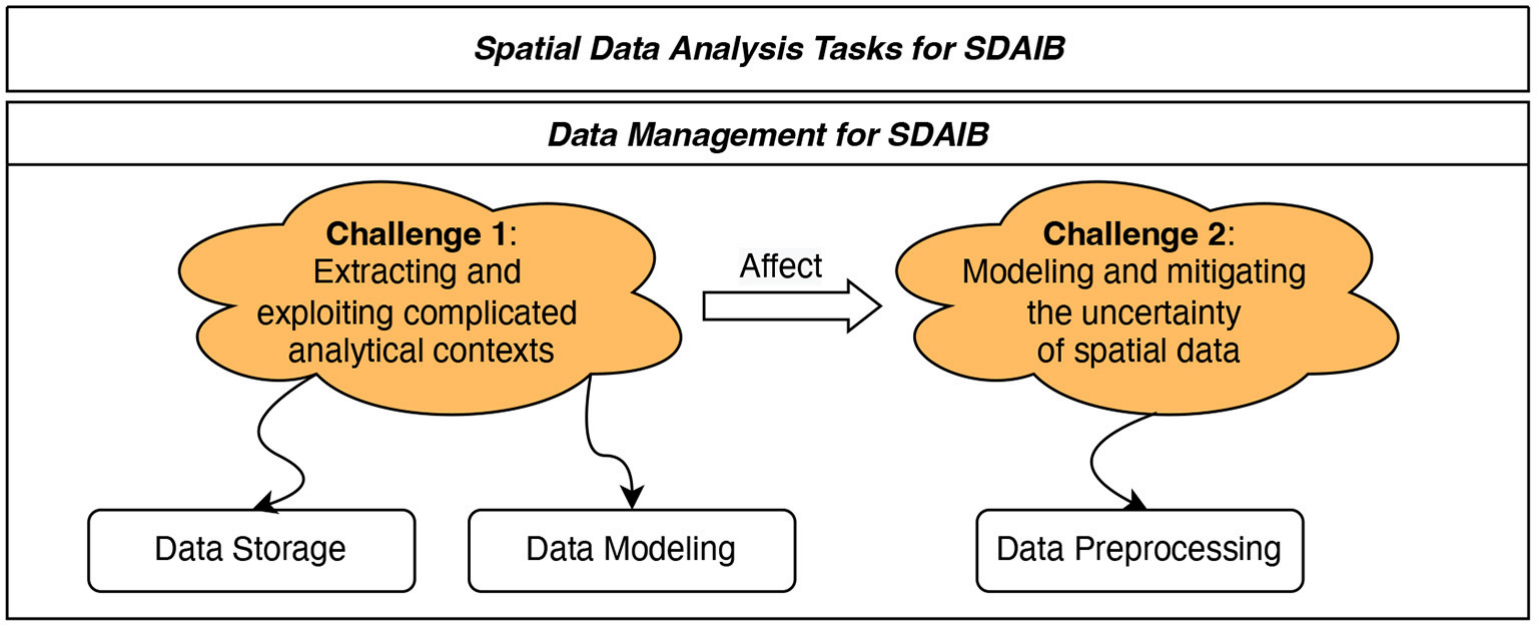
Two general data management challenges in SDAIB.

The past two decades have seen a lot of spatial data analysis research for different building types and different application scenarios (cf. literature reviews Cheema, 2018; Qolomany et al., 2019; Daissaoui et al., 2020). Setting aside those application-specific technical difficulties, we identify two general challenges faced by SDAIB in regard to the fundamental data management, as presented in Figure 2.

- **Extracting and exploiting complicated analytical contexts**. In most SDAIB tasks, algorithms are required to be aware of the analytical context for extracting accurate knowledge and making correct decisions accordingly. For example, in the distance monitoring task, physical contexts such as windows, obstacles, and floors must be taken into account in the distance estimation. Otherwise, the obtained results are inaccurate or even useless (Yang et al., 2010; Chan et al., 2022). For another example, in the path planning task, if the algorithm can obtain real-time and accurate contextual information about the populations and flows in each room, it can find for users or robots the routes that avoid potential congestion and collisions—such routes are practically more useful than those routes with the shortest distances (Liu et al., 2021c). However, the analytical contexts in SDAIB are complicated and diverse, involving many aspects related to the building environment and the internal entities like human beings and sensory devices. Many existing works (Yang et al., 2010; Kamkarian and Hexmoor, 2012; Lin et al., 2016; Teng et al., 2017; Li et al., 2018c, 2020b; Liu et al., 2021c) find relevant analytical contexts according to the need of the analytical task and design specific data structures for them. In this light, it is desirable to have a general mechanism for efficiently and effectively modeling different types of analytical contexts, in order to facilitate the data modeling and data storage for SDAIB tasks.
- **Modeling and mitigating the uncertainty of spatial data**. Indoor positioning technologies and other sensing technologies (such as Bluetooth- and RFID-based tracking) typically offer limited accuracy and low sampling rates, resulting in low quality of spatial data used for analyses (Liu et al., 2007; Xie et al., 2013; Lu et al., 2016). In particular, the limited accuracy makes an object's observed location deviate, while the low sampling rate makes the object often unobserved. Both two issues enlarge the uncertainty of spatial data and thus lower the effectiveness of downstream SDAIB tasks. To make matters worse, buildings involve much more complicated analytical contexts in relatively small spaces compared to outdoors, further amplifying the negative effects of spatial data uncertainty. For example, in the semantic trajectory generation task, multiple indoor regions, such as shops in a mall, are close to each other and are only segmented by walls, and a positioning deviation of merely a few meters will likely cause a mis-annotation of the target object's located region (Li et al., 2020a,b). In the hotspot discovery task, the unique indoor topology and the low-sampling issue incur multiple possible paths for a moving object, which complicate the computation and reduce the accuracy of the computation results (Li et al., 2018b). Modeling and reducing spatial data uncertainty have been widely studied for Euclidean space and road networks (Züfle, 2021). However, those data preprocessing techniques fall short in the intelligent building setting that is characterized by the unique indoor topology and indoor positioning issues. Notably, addressing the challenges of spatial data uncertainty relies on appropriate handling of the analytical contexts (see Figure 2). This is because the contexts involved in intelligent buildings provide key prior knowledge for modeling and reducing spatial data uncertainty; some examples have been provided later in Section 2.2.3. Therefore, it is considered a proper routine to model the context first and then the spatial data uncertainty.

In this paper, we will introduce the recent progress in addressing these two general challenges in SDAIB. To be specific, Section 2 will review the recent efforts to handle analytical contexts and spatial data uncertainties for SDAIB, respectively. Based on a classification of the existing works and a summary of their technical highlights, we will propose a unified framework for accommodating modeling techniques for a variety of analytical contexts and spatial data uncertainties in Section 3. Subsequently, Section 4 will select four representative SDAIB tasks and demonstrate the usage of the proposed modeling framework for context-aware and uncertainty-aware SDAIB. The four tasks are hotspot discovery, path planning, semantic trajectory generation, and distance monitoring. In Section 5, we will discuss the open issues and emerging opportunities of SDAIB in the new technology ecosystem. We will finally conclude the paper in Section 6.

## 2. Modeling techniques in SDAIB

We will revisit and summarize relevant techniques for modeling analytical contexts and spatial data uncertainties in Sections 2.1 and 2.2, respectively.

### 2.1. Modeling analytical contexts

**Figure 3:**
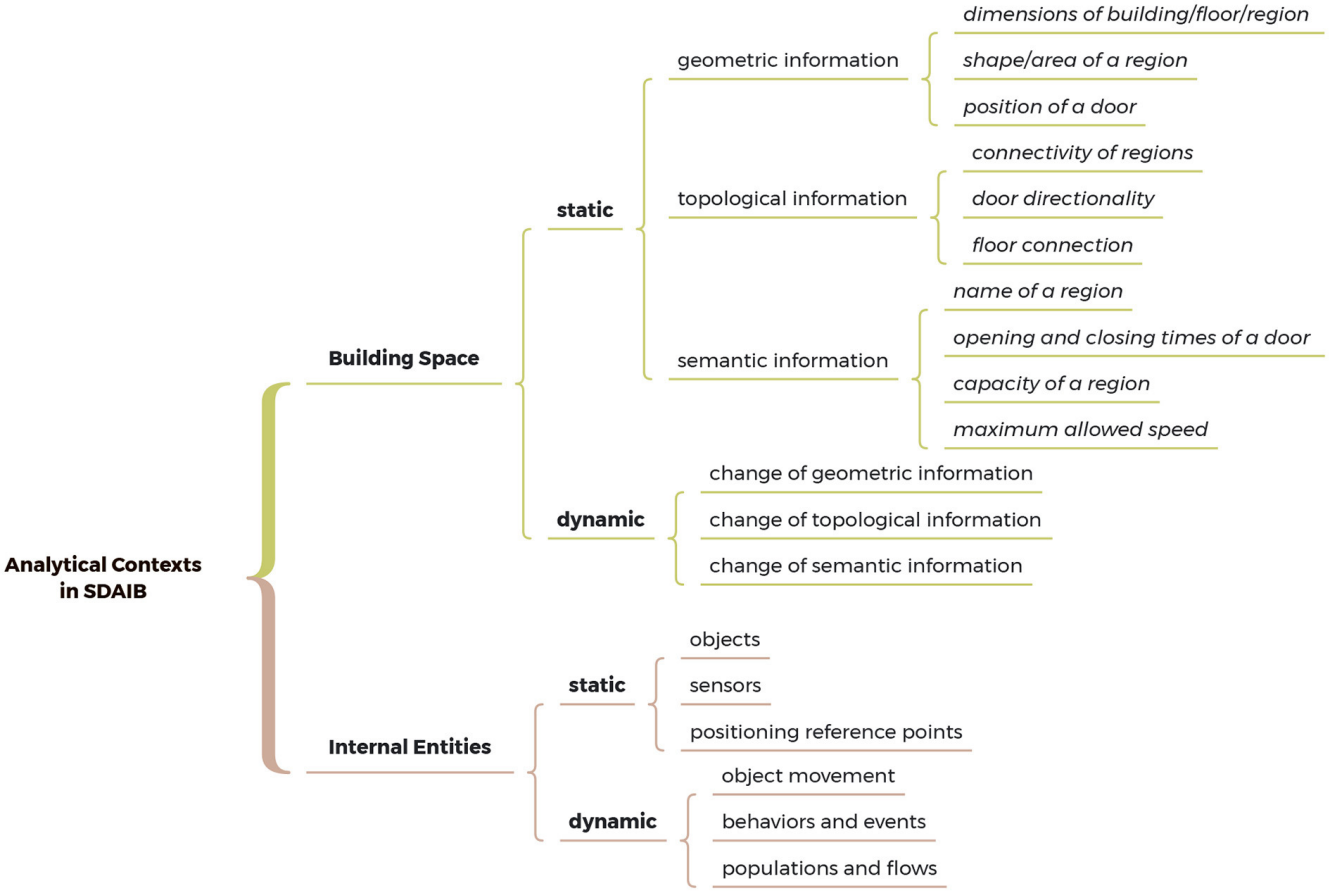
A non-exhaustive taxonomy of the analytical contexts in SDAIB.

The analytical contexts refer to a set of important data that exists along with the analytical process, describing the states of related objects in processing, the ancillary information, the requirements of the analysis task, etc. Figure 3 presents a non-exhaustive taxonomy of the analytical contexts in SDAIB, in which we categorize analytical contexts into the contexts related to the building space and those related to the internal entities in the building. Note that we exclude the human-centered contexts (e.g., user preference and sentiment) because they are considered not specific to spatial data analyses. In each category, we further distinguish the *dynamic (analytical) contexts* from those static contexts. The former evolves with the analytical process while the latter does not. We proceed to go through different kinds of analytical contexts in SDAIB and describe their scope and recent efforts in handling them.

#### 2.1.1. Building space

##### 2.1.1.1. Static contexts of building space

The building environment, whose geometric information (e.g., the dimensions of the whole building or a region, and the position of a particular door), topological information (e.g., the connectivity or accessibility between adjacent regions and floors), and semantic information (e.g., the name of a region and the maximum capacity of a region), are all fundamental to SDAIB (Worboys, 2011). Therefore, building space (or indoor space) models are usually hybrid, in which the basic data structure (Afyouni et al., 2012) is constructed based on either of the three aspects whereas the other two are extended on the basic data structure.

To represent two- or three-dimensional *geometric information* of the *building elements* (e.g., doors, windows, rooms, and staircases), the lattices (Elfes, 1989; Li and Lee, 2008; Lin et al., 2017), tetrahedrons (Penninga et al., 2006), polygonal prisms (Kim et al., 2009), Voronoi tessellation (Choset and Burdick, 2000), boundary-based representation (Crowley, 1989) have been exploited, on top of which tree-based (Li and Lee, 2008) or graph-based (Penninga et al., 2006; Kim et al., 2009) indexes can be built to accelerate spatial search.

*Topological information* captures the relationship between the building elements, for which the graph structure (Lee, 2004) is the most commonly used. Typically, *building partitions* (e.g., a room or a region) are represented by graph nodes, while their interactions, such as adjacency and intersection (Lee, 2004; Kim et al., 2009; Lin et al., 2017), mutual visibility Franz et al. (2005), connecting doors (Lu et al., 2012; Xie et al., 2014), passed sensors (Jensen et al., 2009a; Teng et al., 2017), passed positioning reference points (Li et al., 2018b), are recorded at graph edges. In some settings, considering the search efficiency, a dual transformation of the above structure is applied. For example, a *door graph* (Yang et al., 2010) with doors as nodes and spatial information between doors as edges is used to facilitate the calculation of the minimum indoor walking distance; a *deployment graph* (Baba et al., 2014) capturing RFID readers as nodes and passed region(s) between two readers as edges is designed for cleaning RFID reader ID sequences. Recently, some work considers indexing topological information to improve computation efficiency, e.g., VIP-tree (Shao et al., 2016) is a hierarchical model on the connectivity of rooms to speed up the shortest path finding.

*Semantic information* is indispensable in many indoor location-based services, such as navigation (Li and Lee, 2008; Lin et al., 2013; Kang and Li, 2017) and door access control (Bhatt et al., 2009). Pure semantic models for buildings are often object-oriented, using UML-like languages [such as CityGML (Kolbe et al., 2005) and indoorGML (Kang and Li, 2017)], task-specific ontologies (Bhatt et al., 2009; Wu et al., 2018), IFC (International Foundation Class) data formats (Lin et al., 2013), etc. It is worth noting that semantic information is often associated with the building elements, e.g., names and functionality of rooms. Therefore, compared to maintaining a purely object-oriented semantic model, it would be more efficient to attach the semantic information to the building's spatial model which can be easily enhanced with a spatial index, as done in many existing works (Li and Lee, 2008; Teng et al., 2017; Li et al., 2018b, 2020a; Feng et al., 2020; Liu et al., 2021a).

##### 2.1.1.2. Dynamic contexts of building space

Since the building environment is dynamic, all three types of information described above may evolve during the analytical process. Relatively few studies have considered dynamic modeling of building space information:

- *Change of geometric information*. To capture the change in the geometric information of the building elements, an event-based updating approach is often used, i.e., deleting outdated information and inserting new information. Correspondingly, the spatial index should be updated incrementally or fully (Li and Lee, 2008; Lu et al., 2012; Xie et al., 2014; Liu et al., 2021b). Since the geometric information changes infrequently, event-based updating is acceptable for most applications.
- *Change of topological information*. The topological relationship between building elements should be updated timely when particular events happen, such as the lock and unlock of a door, the combination or division of building partitions, and the block of a region. Otherwise, such changes may invalidate the computation results in services like real-time indoor navigation (Liu et al., 2021a). Walton and Worboys (2012) model the static topological information using a bi-graphical model. In their topological model, the dynamic change is defined by a reaction rule consisting of a pair of a pattern to be changed and a resulting pattern. Xie et al. (2014) maintain for each building partition a set of pointers to other partitions, which can be updated dynamically. Also assuming a graph-based model, Liu et al. (2021a) propose both synchronous and asynchronous mechanisms to check the temporal variations of door's accessibility along the path planning process. This work also utilizes VIP-tree (Shao et al., 2016) to index the topological information. To reflect the topological changes on the index, life span interval information is extended to the internal nodes of the tree.
- *Change of semantic information*. For most models that augment semantic information to a base spatial model, the event-based updating approach and the life span interval definition can handle the dynamic changes of building semantics. Recently, some studies (Teng et al., 2018; Guo et al., 2021) consider extracting updates of semantics from crowdsourced images and videos for building self-updating indoor semantic floorplan models.

#### 2.1.2. Internal entities

The internal entities that are interesting to SDAIB include, but are not limited to, stationary objects (e.g., appliances, furniture, obstacles, and indoor POIs), moving objects (e.g., pedestrians, robots, and vehicles), sensors (e.g., smoke detectors and RFID readers), and positioning reference points^2^.

##### 2.1.2.1. Static contexts of internal entities

For internal entities, the approaches to modeling their static geometric, topological, and semantic information can follow those for the building elements presented in Section 2.1.1. In general, object-oriented models can be used to maintain various aspects of information of each internal entity, including UML-like languages and IFC data formats. For example, IFC data format (Lin et al., 2013) can represent the furnishing objects and facilities in a building. Moreover, for search and storage efficiency, the maintained internal entities are often attached to the existing spatial model of the building space, according to the spatial relationship (e.g., intersection and containment) between the internal entity and the building space. To realize this, pointers, linked lists, and hash tables are used. For example, the composite indoor index (Xie et al., 2014) maintains an object bucket for each of its building partitions and an object hashtable for quickly finding the pointer of the host partition of a particular object; the deployment graph (Jensen et al., 2009a) links doors and partitions to the two-dimensional location, activation range, and types of each deployed RFID reader.

##### 2.1.2.2. Dynamic contexts of internal entities

In practice, internal entities feature dynamic changes. Therefore, a good body of modeling techniques has been proposed to handle different kinds of dynamic contexts related to internal entities. Several important dimensions are introduced below.

###### 2.1.2.2.1. Object movement

For the real-time position changes of moving objects, some works (Li et al., 2018c; Chan et al., 2022) consider updating the mappings between objects and the building partitions to which they belong. However, this mechanism is not suitable for analyzing large-scale historical trajectory data as it may lead to high accumulated processing latency. Therefore, indexing techniques are usually applied to historical trajectories generated by indoor moving objects, either based on R-trees (Jensen et al., 2009b; Alamri et al., 2013; Lin et al., 2016) or grid cells (Choi et al., 2004). Such indexes facilitate the range and nearest neighbor queries, which lay the foundation for other complex spatial computations.

###### 2.1.2.2.2. Behaviors and events

Becker et al. (2009) propose a multilayered space-event model for indoor navigation, in which the sensor space layer is built on top of the topographic space layer for maintaining the locations and ranges of installed transmitters and sensors. Likewise, Jin et al. (2012) design a conceptual modeling framework for indoor space, whose moving object layer supports tracking an indoor entity's visiting durations, moving patterns, and other behavioral information. Kim et al. (2014) pay particular attention to indoor facility management and propose a CityGML extension of modeling indoor facility features. Li et al. (2020a,b) model an indoor behavior as a triple [*s, e*, τ], which captures an object's event *e*∈*E* in a semantic region *s*∈*S* during a particular time period τ.

###### 2.1.2.2.3. Populations and flows

In many tasks, such as path planning (Liu et al., 2021c) and crowd evacuation (Kamkarian and Hexmoor, 2012), algorithms need to know the populations of regions and the flow of doors in a building. To support the planning of pedestrian facilities, Lam et al. (2003) devise a generalized walking time function considering bi-directional flow distributions, which can be calibrated for various flow conditions. Aiming for finding populated locations using RFID-like tracking data, Ahmed et al. (2017) propose a hierarchical location-time index that maintains the number of distinct objects entering, exiting, and presence at a particular location at discrete timestamps or during time intervals. To enable finding a path with the least encountered objects or a path with the shortest travel time in the presence of crowds, Liu et al. (2021c) capture the flows of doors using a Poisson distribution based function.

#### 2.1.3. Summary of modeling techniques for analytical contexts

To sum up, multiple aspects of analytical contexts have been considered and modeled in existing studies. In Table 1, we select representative studies on modeling contexts for SDAIB. We can find that these studies accommodate different modeling techniques for different application goals. However, these works do not consider a common methodology to guide analysts in the design and adoption of different types of modeling techniques, which is indeed the main objective of our work (as to be detailed in Section 3).

**Table 1:** Featural comparison of representative studies on intelligent building context modeling.

**References**	**Building space**						**Internal entities**		**Remarks**
	**SG**	**ST**	**SS**	**DG**	**DT**	**DS**	**Static**	**Dynamic**	
Li and Lee (2008)	✓	–	✓	–	–	–	–	–	Basic semantic relationships like containment and overlap
Worboys (2011)	✓	✓	✓	–	–	–	–	–	Formal models for building space
Jin et al. (2012)	✓	✓	✓	–	–	–	✓	✓	Supports sensors and static/moving objects
Lu et al. (2012)	✓	✓	–	✓	✓	–	–	–	Focuses on distance-related computations in building spaces
Lin et al. (2013)	✓	✓	✓	✓	✓	✓	✓	✓	Support dynamic changes *via* database update
Alamri et al. (2013)	✓	✓	–	✓	✓	–	✓	✓	Support dynamic changes *via* indexing
Xie et al. (2014)	✓	✓	–	✓	✓	–	✓	✓	Support dynamic changes *via* indexing and pointers
Kang and Li (2017)	✓	✓	✓	✓	✓	✓	–	–	Semantic representations of building spaces
**Objective of this work**	✓	✓	✓	✓	✓	✓	✓	✓	Methodology on context modeling

**SG**, static geometric information; **ST**, static topological information; **SS**, static semantic information; **DG**, dynamic geometric information; **DT**, dynamic topological information; **DS**, dynamic semantic information.

Aiming for formulating the methodology of modeling analytical contexts in SDAIB, we draw the following findings from the aforementioned studies. First, spatial models for buildings, whether based on geometric information or topological information, are suitable as the *base* model for organizing the analytical contexts, with which semantic information can be efficiently associated. When discussing a base model here, we mean a basic data structure that can be extended with advanced models/functions for handling specific types of analytical contexts. Second, for dynamic contexts of the building space, amendment and updating to the base model are often needed; in contrast, dynamic changes of the internal entities can be modeled by some external, complex models/functions that are flexibly linked to the base model.

### 2.2. Modeling spatial data uncertainty

**Figure 4:**
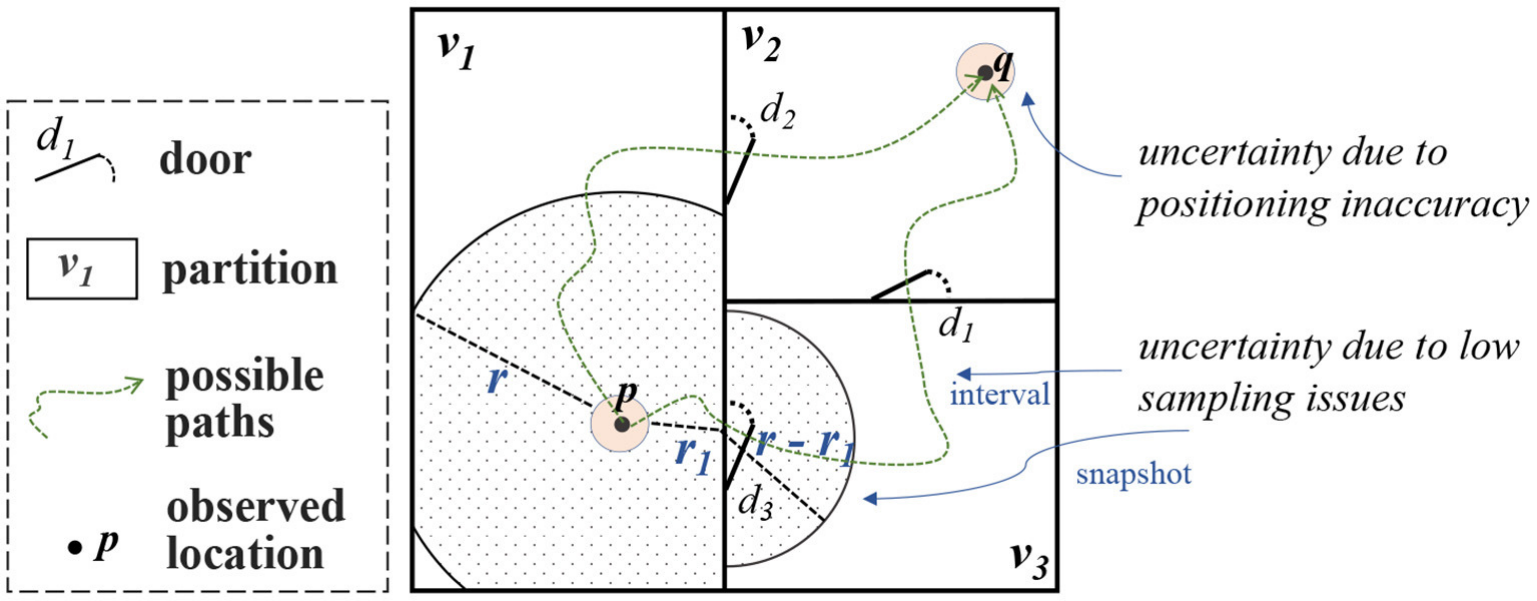
An example of different kinds of spatial data uncertainties in SDAIB.

**Figure 5:**
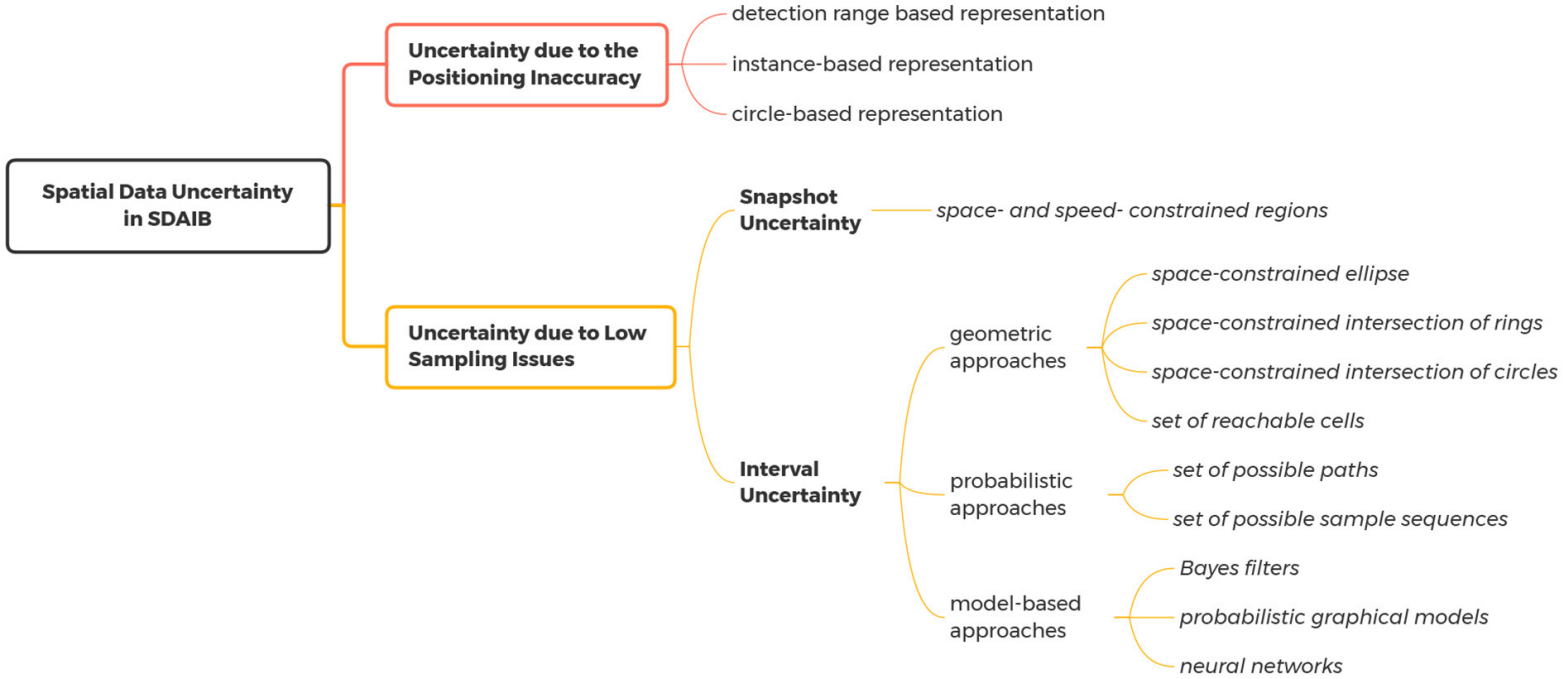
The classification of the approaches for handling spatial data uncertainties in SDAIB.

The spatial data uncertainty in SDAIB is mainly caused by two technical limitations of indoor positioning (tracking) systems, namely (1) the inaccuracy of the positioning algorithm and (2) the discrete sampling scheme of the system. The former is mainly reflected in the errors of estimated locations observed at a specific time point, while the latter results in incomplete and insufficient overall information for analysis. An example is shown in Figure 4. Suppose *p* and *q* are two locations consecutively observed by the positioning system at timestamps *t*_*p*_ and *t*_*q*_, respectively. Due to the positioning inaccuracy, there are many possibilities for the actual locations at *t*_*p*_ and *t*_*q*_. In Figure 4, two small circles in pink are used to indicate the spatial uncertainties caused by positioning inaccuracy. On the other hand, the object whereabouts between *t*_*p*_ and *t*_*q*_ also have many possibilities as there are no observations in-between: First, the snapshot location at time *t* ∈ (*t*_*p*_, *t*_*q*_) is undetermined (called *snapshot uncertainty*) but should be constrained within a geometric region, e.g., as indicated by the shaded portions in Figure 4, the location at *t* should be within an indoor distance *r* to the location at *t*_*p*_ such that *r* is the maximum distance one can reach from *t*_*p*_ to *t*; Second, the overall movement from *t*_*p*_ to *t*_*q*_ is also unknown (called *interval uncertainty*), e.g., one can reach *q* from *p* through either the door *d*_2_ or the doors *d*_3_ and *d*_1_, as illustrated by the two possible paths in Figure 4. In general, the modeling techniques for the uncertainties due to the positioning inaccuracy and those due to low sampling issues are quite different. As such, we present a classification of relevant techniques in Figure 5. Their details are given below.

#### 2.2.1. Uncertainty due to the positioning inaccuracy

For a target object at a particular timestamp, the estimated location may deviate from the true location due to the inherent positioning error. This phenomenon is reflected in the uncertainty of the observed spatial data (Hunter, 1999; Pfoser and Jensen, 1999; Jeung et al., 2013; Züfle et al., 2017), which must be taken into consideration in spatial data analyses, especially for the indoor scenes that usually pose high requirements on the precision of the spatial data.

Different modeling approaches have been employed, depending on the technologies (e.g., Wi-Fi, RFID, or Bluetooth) and positioning algorithms (e.g., proximity-based, geometry-based, or learning-based) employed in the indoor positioning system (Liu et al., 2007).

In the proximity-based positioning that is commonly seen in RFID- and Bluetooth-based tracking solutions, the *activation range based representation* (Jensen et al., 2009a; Yang et al., 2010; Lu et al., 2011, 2016) is employed to model the uncertainty of the object location reported in a tracking record. Specifically, a tracking record (*o*_*i*_, *r*_*j*_, *t*) indicates that an object (e.g., an RFID tag) *o*_*i*_ has been detected by a device (e.g., an RFID reader) *r*_*j*_ at time *t*. As the proximity-based tracking cannot give exact geometric coordinates, the possible position of *o*_*i*_ at *t* is simply considered to be within the activation range of the device *r*_*j*_, which is usually modeled as a small circle °(*l*_*j*_, *mrd*_*j*_), centered at *r*_*j*_'s deployment position *l*_*j*_ with the maximum reader distance *mrd*_*j*_ of *r*_*j*_ as the radius.

The location result as a geometric coordinate is more widely used in practice (Liu et al., 2007). Its form of (*o*_*i*_, *l*_*j*_, *t*) indicates that object *o*_*i*_ is considered to be at a specific indoor location *l*_*j*_ at time *t*. Usually, the indoor location is a two-dimensional plane point on a particular floor. In this study, for the sake of presentation simplicity, we focus on the uncertainty of the estimated location on a determined floor as the floor determination has proven highly accurate (>99%; Wang and Luo, 2017). For geometric positioning results, the two following kinds of representations are often used.

- The *instance-based representation* (Xie et al., 2013, 2014; Li et al., 2018b) models the possible locations of the target object as a set of location samples, each being a pair of a location and an existence probability. Formally, the sample set is denoted as {(*l*_*i*_, ρ_*i*_)} where *l*_*i*_ is the sample location and ρ_*i*_ is the corresponding probability such that $\underset{i}{\sum }\rho_{i}=1$. The instance-based representation fits well with typical localization algorithms, such as *k*NN and naive Bayes (Liu et al., 2007), which produce multiple possible locations along with their corresponding likelihoods. Moreover, instances can be used to approximate arbitrary distributions, providing high flexibility.
- The *circle-based representation* (Chan et al., 2022) captures the possible location of the target object *o*_*i*_ as a circle °(*l*_*j*_, ϵ) where the circle center *l*_*j*_ is the observed location and the radius ϵ is the pre-known average positioning error of the underlying positioning system. The circle-based representation can be modified and extended with prior knowledge, such as using different radii at different locations (Chan et al., 2022). It can also be converted into the instance-based representation (Chan et al., 2022). However, it is nontrivial to determine the radii at different building locations.

#### 2.2.2. Uncertainty due to low sampling issues

For the uncertainty caused by low sampling issues, we further differentiate the *snapshot uncertainty* and the *interval uncertainty*. Their main difference is that the snapshot uncertainty discusses the object's whereabouts at a particular time point, while the interval uncertainty concerns the object's whereabouts during a time interval between two observations (Lu et al., 2016).

##### 2.2.2.1. Snapshot uncertainty

Snapshot uncertainty is caused when the last observation has expired while the current object location has not been observed. Given the latest positioning record (*o*_*i*_, *x*_*j*_, *t*_*l*_), where *x*_*j*_ is the observed location at the latest sampling time *t*_*l*_, the possible locations of *o*_*i*_ at the current time *t*_*c*_(>*t*_*l*_) can be derived based on *geometric approaches* (Yang et al., 2010; Li et al., 2018c; Chan et al., 2022), as a space- and speed-constrained region. To be specific, given the maximum speed *V*_*max*_ of all indoor moving objects^3^, the possible locations of the object *o*_*i*_ at time *t*_*c*_ are covered by an *uncertainty region* *UR*(*x*_*j*_, *t*_*l*_, *t*_*c*_), which is computed as follows.


(1) $$UR(x_{j},t_{l},t_{c})=\left\{ \begin{array}{l} {\text{Range}}_{I}(x_{j},\delta ),\text{if}\;x_{j}\;\text{is a specific location},\\ \cup_{p\in x_{j}.\text{boundary}}{\text{Range}}_{I}(p,\delta )\setminus x_{j},\text{if}\;x_{j}\;\text{is an}\\ \text{activation range},\\ \cup_{e\in x_{j}}{\text{Range}}_{I}(e,\delta ),\text{if}\;x_{j}\;\text{is an instance set},\\ \cup_{p\in x_{j}.\text{boundary}}{\text{Range}}_{I}(p,\delta )\cup x_{j},\text{if}\;x_{j}\;\text{is a}\\ \text{small circle}. \end{array}\right.$$

where δ = *V*_*max*_ · (*t*_*c*_ − *t*_*l*_) is the maximum distance that *o*_*i*_ can walk from *t*_*l*_ to *t*_*c*_, and the Indoor Range Query function Range_*I*_(*p, dis*) returns the indoor portions that can be reached within the distance *dis* from the given point *p*. The implementation of the Range_*I*_ function has been covered in existing studies (Lu et al., 2012; Xie et al., 2013; Li et al., 2018c). In Equation (1), Case 1 assumes there is no positioning uncertainty (Li et al., 2018c), while the other three cases correspond to different kinds of representations of positioning uncertainties (see Section 2.2.1). In particular, Case 2 considers the activation range based representation (Yang et al., 2010) and *x*_*j*_ is excluded because the object is currently unobserved and must not be within any activation range; Case 3 considers the instance-based representation, and each instance is used to infer an uncertainty region that will be merged then; Case 4 refers to the circle-based representation (Chan et al., 2022) used in geometric positioning and the uncertainty region is modeled as an outwardly extended region of the last observed location *x*_*j*_.

Generally speaking, the distribution of possible locations in the derived uncertainty region is not necessarily uniform. Following the first law of geography (Tobler, 2004), i.e., things that are near are more related than things that are far away, various *distance-decaying functions* (Li et al., 2018c; Chan et al., 2022) have been designed to model the likelihood of different possible locations in an uncertainty region.

##### 2.2.2.2. Interval uncertainty

Unlike snapshot uncertainty, interval uncertainty happens across the intervals between two consecutively observed locations. We categorize three different types of approaches for modeling interval uncertainty as follows.

- The *geometric approaches* (Jensen et al., 2009a; Lu et al., 2011, 2016; Teng et al., 2017; Li et al., 2020b) calculate the possible locations of an object between its two consecutively observed locations based on spatial constraints. Suppose (*x*_*a*_, *t*_*a*_) and (*x*_*b*_, *t*_*b*_) are two consecutively positioning records, where *x*_*a*_ and *x*_*b*_ are observed locations and *t*_*a*_ and *t*_*b*_ are the corresponding timestamps. Referring to Section 2.2.1, an observed location *x*_*a*_ in the RFID setting is represented as a small circle °(*l*_*a*_, *mrd*_*a*_) centered at the reader's deployed location *l*_*a*_ with the maximum reader distance *mrd*_*a*_ as the radius. Jensen et al. (2009a) and Lu et al. (2016) point out that the possible locations during the time interval [*t*_*a*_, *t*_*b*_] must be constrained by an extended ellipse, whose two foci are located at *l*_*a*_ and *l*_*b*_ and eccentricity is defined by the distance [*V*_*max*_ · (*t*_*b*_ − *t*_*a*_) + *mrd*_*a*_ + *mrd*_*b*_] where *V*_*max*_ is the maximum speed of moving objects. Moreover, the ellipse must exclude those unreachable indoor parts in terms of minimum indoor walking distance, rather than the Euclidean distance. Also in the RFID tracking scenario, Lu et al. (2011) indicate that the possible location at a particular time point *t*_*c*_ ∈ [*t*_*a*_, *t*_*b*_] is constrained by two rings ring[*l*_*a*_, *mrd*_*a*_, *V*_*max*_ · (*t*_*c*_ − *t*_*a*_)] and ring[*l*_*b*_, *mrd*_*b*_, *V*_*max*_ · (*t*_*b*_ − *t*_*c*_)], where ring(*p, dia*_1_, *dist*_2_) refers to a ring centered at point *p* with inside diameter *dia*_1_ and outside diameter *dia*_2_. Finally, the intersection of the two rings excluded those unreachable indoor portions is used to model the uncertainty region at time *t*_*c*_. Similar to the space-constrained intersection of rings (Lu et al., 2011), Li et al. (2020b) employ the space-constrained intersection of circles for geometric positioning results. Assuming grid-based partitioning of the target indoor space, Teng et al. (2017) use a set of reachable cells to represent possible object locations. Each grid satisfies that any moving object can reach the previously observed location *l*_*a*_ and next observed location *l*_*b*_ within the limited traveling time.
- The *probabilistic approaches* (Li et al., 2018b, 2020b) represent the possible indoor movement between two observed locations in a probabilistic form. Representing the indoor space as a graph with rooms as nodes and doors as edges, Li et al. (2020b) compute the transition probability on each directed edge using historical data, and analyze the movement between two observation locations (rooms) based on conditional probabilities with the computed transition probabilities as prior knowledge. Assuming that the location at each observed time point is a set of instances with probabilities, another work (Li et al., 2018b) uses the Cartesian product of the instances at continuous time points to generate a set of possible indoor paths, and the probability of each path is calculated as the product of the probabilities of the instances it goes through. Those indoor paths violating indoor topology will be removed and the probabilities of the rest paths will be normalized.
- The *model-based approaches* (Hightower and Borriello, 2004; Petzold et al., 2005; Bekkali et al., 2007; Asahara et al., 2011; Laursen et al., 2012; Patel and Thakore, 2013; Yu et al., 2013; Baba et al., 2016; Belmonte-Hernandez et al., 2019; Li et al., 2020a; Tariq et al., 2021) capture the underlying data generation mechanism of the observed data in a model, which is then used to recover the unsampled time points. Three representative techniques, namely Bayes filters, probabilistic graphical models, and neural networks, have been explored, mainly depending on capturing the temporal dependencies of the sequential positioning records. The Bayes filter sequentially estimates the optimal location of the target object by mapping the noisy location observations at each time point to a fuzzy representation, such as samples in particle filters (Hightower and Borriello, 2004; Yu et al., 2013) and Gaussian distributions in Kalman filters (Bekkali et al., 2007; Patel and Thakore, 2013). Probabilistic graphical models represent the observed object positions as discrete and piecewise constant states and learn dependencies between those states using historical data. Different models have been explored to incorporate different kinds of application semantics, such as hidden Markov models (Laursen et al., 2012; Baba et al., 2016), conditional random fields (Li et al., 2020a), dynamic Bayesian networks (Petzold et al., 2005), and mixed Markov-chain models (Asahara et al., 2011). Recently, several works propose using deep neural networks to learn intrinsic dependencies among sequential positioning records, such as recurrent neural networks (Belmonte-Hernandez et al., 2019) and 1-D convolutional networks (Tariq et al., 2021).

#### 2.2.3. Summary of modeling techniques for spatial data uncertainties

First, to handle the spatial data uncertainty, the characteristics of the indoor positioning/tracking system, such as the used technologies and positioning algorithms, must be taken into account. Second, the uncertainty due to the positioning inaccuracy should be handled before the uncertainty due to low sampling issues. In the latter case, the handling techniques usually utilize spatial observations to derive the uncertainty regions. Such spatial observations, obtained from an indoor positioning system, often carry positioning errors (see Section 2.2.1). As these positioning errors take effect in the analyses, they should be handled before deriving the uncertainty regions with respect to low sampling issues. Referring to the example in Figure 4, the positioning errors of the observed location *p* are considered in modeling the snapshot uncertainty at a time *t* ∈ (*t*_*p*_, *t*_*q*_) (the shaded regions). Last, in handling uncertainties caused by low sampling, the analytical contexts are very useful. For example, the Indoor Range Query function used in snapshot uncertainty models (Xie et al., 2013; Li et al., 2018c) heavily relies on the geometric and topological information of the building space: To return the indoor portions that one can reach within a limited time budget, the function needs to know the positions of doors to access adjacent rooms as well as the size of rooms. In Figure 4, deriving the uncertainty region (the shaded portions) considers the position of door *d*_3_ and geometries of partitions *v*_1_ and *v*_3_. For another example, some model-based approaches for interval uncertainties (Laursen et al., 2012; Yu et al., 2013; Li et al., 2020a) require both spatial and semantic information to build a probabilistic graphical model. In particular, semantics like sensor deployment and geometries of indoor space are used as prior knowledge for designing the corresponding state space.

## 3. A unified modeling framework

**Figure 6:**
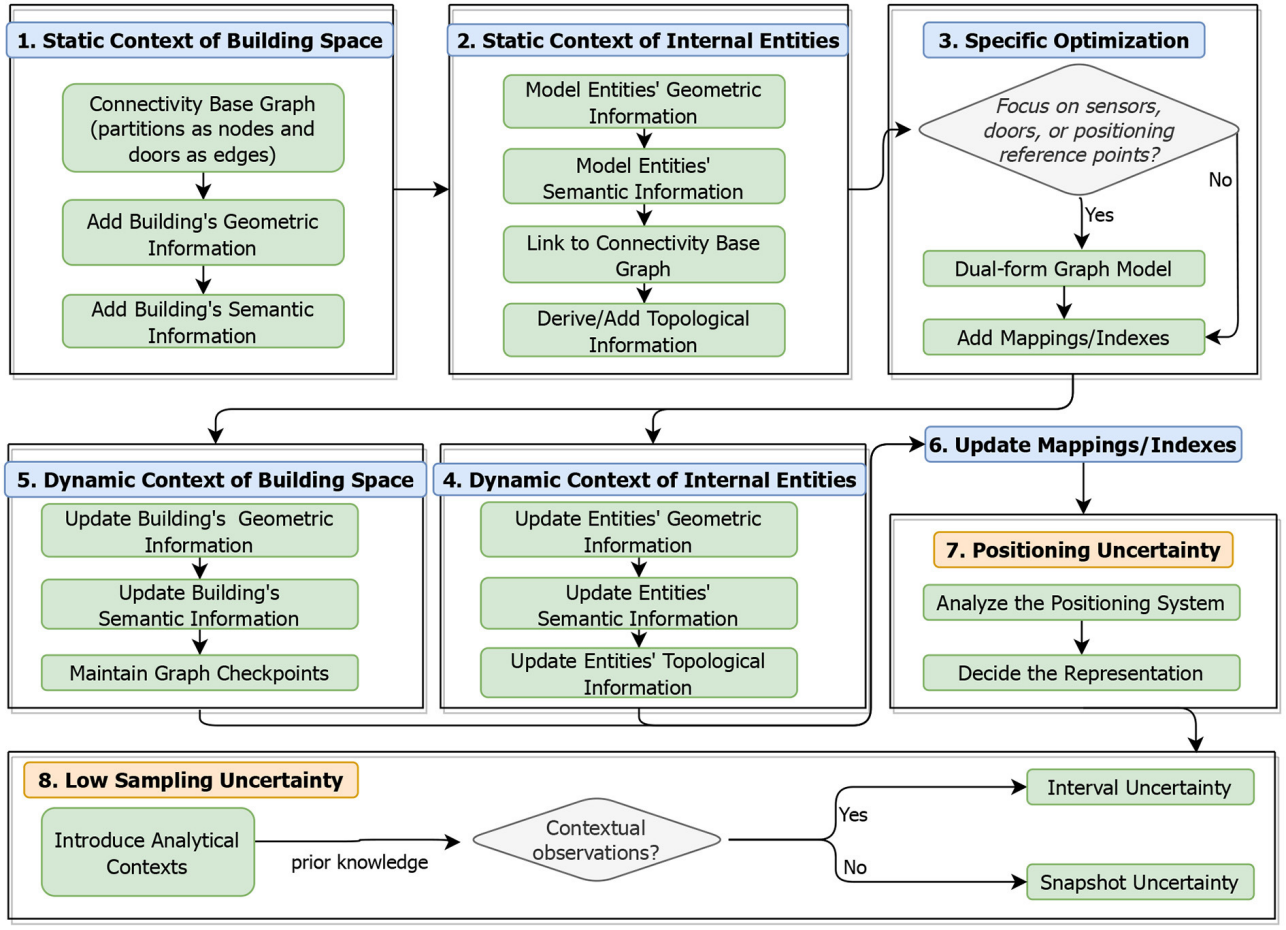
A unified framework for modeling analytical contexts and spatial data uncertainties.

Given that various kinds of analytical contexts and spatial data uncertainties should be considered in different SDAIB tasks, we propose a unified modeling framework based on which data analysts can incorporate relevant contexts and spatial data uncertainties into their specific tasks through a standard workflow. The framework is illustrated in Figure 6, which consists of six steps for modeling analytical contexts followed by two steps for modeling spatial data uncertainties. We model the analytical contexts first and then spatial data uncertainties because handling data uncertainties usually rely on the contexts.

1. **Handling Static Contexts of Building Space**. According to the summary in Section 2.1.3, we first use the spatial information (including geometric and topological information) to build a base model called *connectivity base graph* (Jensen et al., 2009a)^4^. We use a graph-based model due to its flexibility in handling dynamic changes in both nodes and edges. Specifically, we divide the target building space into a set of basic topological units according to the geometric decomposition algorithm (Xie et al., 2014). Each such unit is called a partition and represented as a graph node. Then, each two topologically connected partitions are represented by an edge, on which the connecting door is maintained. Using directed edges is an alternative solution if the door directionality is critical (see *accessibility graph*; Jensen et al., 2009a). Afterwards, we capture the geometric and semantic properties of each building element using object-oriented data models and link those data models to the components of the connectivity base graph (i.e., doors and partitions).
2. **Handling Static Contexts of Internal Entities**. Similar to linking building elements to the connectivity base graph, internal entities such as sensors and positioning reference points are associated with the graph nodes and edges after being modeled with geometric and semantic properties. Some analyses require knowing topological relationships between specific internal entities. For example, the asset tracing task may need to know whether two specific RFID readers' activation ranges overlap with each other (Jensen et al., 2009a; Yang et al., 2010; Lu et al., 2011, 2016). Thus, we derive topological relationships of internal entities based on their geometric and semantic information, and add them to the base graph.
3. **Specific Optimization to Static Context Modeling**. To improve data access efficiency for specific tasks, facilitator data structures are designed. Firstly, if the analysis task focuses on the data captured as IDs of sensors (or doors, or positioning reference points), a dual-form transformation of the original connectivity base graph will be beneficial. In particular, the dual-form graph captures the sensors (or doors, or positioning reference points) of interest as graph nodes and their relationships as edges. Secondly, materialized mappings between different kinds of objects can be pre-computed to facilitate search. Moreover, spatial indexes would speed up spatial computations (Liu et al., 2021b), e.g., R-trees and grids for geometric computations (Choi et al., 2004; Lu et al., 2012) and VIP-trees for topology-relevant operations (Shao et al., 2016). Nevertheless, building these additional facilitator data structures incurs more storage space.
4. **Handling Dynamic Contexts of Internal Entities**. Steps 4 and 5 can be performed simultaneously, while Step 4 for internal entities is more straightforward. Specifically, for the dynamic changes of an internal entity, we update its corresponding object-oriented model directly. The update order is geometric information first, semantic information second, and topological information last, considering that the update of one kind of information will affect another kind of information.
5. **Handling Dynamic Contexts of Building Space**. Updating building elements in this step is almost the same as updating internal entities in Step 4, except for the topological information. Since topological relationships between building elements are maintained directly in the base graph, frequently updating the base graph will lead to chained changes. An optimization strategy is to establish checkpoints of the base graph (or its dual-form graph) along the time and maintain incremental updates of graph edges. We refer interested readers to materials (Mondal and Deshpande, 2012; Zaki et al., 2016) on dynamic graph modeling.
6. **Update Mappings/Indexes Related to Dynamic Contexts**. Mappings and indexes should be updated accordingly if there are dynamic information changes in building elements and internal entities. Updating such facilitator data structures will potentially incur a large time overhead, which should be considered carefully in their design. We refer interested readers to an empirical study on indoor spatial indexes (Liu et al., 2021b).
7. **Handling Uncertainty Caused by the Positioning Inaccuracy**. The summary in Section 2.2.3 discloses that positioning uncertainty must be handled before the low sampling uncertainty. To model positioning uncertainty, the representation choice depends on the technology and positioning algorithm employed by the underlying indoor positioning system. Usually, activation range based representation is used in symbolic tracking using RFID and infrared, instance-based representation fits well with the probabilistic positioning algorithms, and circle-based representation is a default choice for geometric positioning results.
8. **Handling Uncertainty Caused by Low Sampling Issues**. Finally, we deal with low sampling uncertainty. Before modeling, we need to introduce relevant analytical contexts as prior knowledge, such as topology and geometric information of the building. If the data observations before and after the analysis time are available, modeling interval uncertainty is considered. Otherwise, we model the snapshot uncertainty. The choice of the modeling approach for low sampling uncertainties (see Section 2.2.2) should consider the characteristics of the indoor positioning system, as well as the availability of prior knowledge and historical positioning data.

The last two steps of the proposed framework focus on modeling data uncertainties related to data analyses, which is considered a fundamental process for reducing data uncertainties in downstream applications. On the one hand, interpolation and sampling techniques on top of a data uncertainty model can be employed to derive or recover the unobserved data (Li et al., 2020b), thus reducing the data uncertainty explicitly. On the other hand, data uncertainty models can be incorporated into some analytical processes [e.g., graphical models Li et al. (2020a) and probabilistic queries Li et al. (2018c)] in a probabilistic way, mitigating the negative impact of spatial data uncertainty in analyses. Examples of reducing data uncertainty based on the modeling techniques will be provided in Section 4.

## 4. Context-aware and uncertainty-aware data analyses

We will describe how the unified modeling framework proposed in Section 3 enables context-aware and uncertainty-aware data analyses. Sections 4.1–4.4 cover hotspots discovery, path planning, semantic trajectory generation, and distance monitoring, respectively. For each task, we will introduce its problem background, modeling process, and analysis algorithms based on the aforementioned framework. We refer interested readers to the original papers for detailed empirical studies.

### 4.1. Hotspots discovery

Public indoor venues, such as shopping malls, subway stations, and airports, are often crowded during peak times. For example, as early as 2004, the weekend traffic at Hong Kong New Town Plaza^5^ reached 320,000 (Xie et al., 2014). Crowd gatherings can easily lead to congestion, safety incidents, and public health concerns. Therefore, it becomes fundamentally important to find out and monitor those indoor hotspots with high populations and flows. Below, we present our techniques for finding out the *indoor dense regions* with the highest populations (Li et al., 2018c) and the *indoor popular semantic locations* with the highest flows (Li et al., 2018b), respectively.

#### 4.1.1. Indoor dense regions

The previous study (Li et al., 2018c) aims to timely find the *k* most dense regions using only the latest geometric positioning result of each observed object in a Wi-Fi based indoor positioning system. The candidate regions are arbitrary rectangles customized by users. As the latest observed locations may be outdated, the counting of objects in a region is based on the objects' uncertainty regions derived from their latest observations. Specifically, for each object *o*_*i*_ whose uncertainty region at the current query time intersects with the candidate region *r*_*j*_, the proportion of the intersecting area occupying *o*_*i*_'s uncertainty region is added to the density of *r*_*j*_ as the probability that *o*_*i*_ currently presents in *r*_*j*_.

The modeling for analytical contexts and spatial data uncertainties are as follows.

- **Analytical Contexts**. For static contexts, a connectivity base graph is built with geometric and topological information of rooms, hallways, and staircases. The indoor moving objects, as internal entities, are maintained in each partition's associated bucket. An R-tree and the mappings between moving objects and partitions are used to speed up locating objects. There is no dynamic change of the building space but the object movements are updated in the base graph and facilitator structures.
- **Spatial Data Uncertainties**. For simplicity, positioning uncertainty is not considered in the latest positioning records—nevertheless, it can be extended to instance-based or circle-based representation. Subsequently, to infer the possible location of an object at a particular query time, the snapshot uncertainty model is introduced, corresponding to Case 1 in Equation (1).

Given the snapshot uncertainty region, it is however time-consuming to compute the probability-based object presence for each pair of a moving object and a candidate region. We briefly introduce the basic idea for efficiently finding the top-*k* densest regions. In particular, instead of calculating the intersecting areas in the indoor space, a simple counting of those certainly and possibly intersected objects is used to derive the lower bound and upper bound of a region's density. In this way, some unpromising regions can be pruned before concrete density computations. The search further utilizes spatial loose bounds enabled by Euclidean distances and temporal loose bounds enabled by the oldest observed timestamps for even more aggressive pruning.

#### 4.1.2. Indoor popular semantic locations

The study (Li et al., 2018c) focuses on identifying those region-like semantic locations with the highest flows during a past time interval. The available historical positioning data is in the instance-based representation and each instance corresponds to a pre-selected reference point in the Wi-Fi based positioning system. Due to low sampling uncertainty, it is necessary to obtain all possible indoor paths of each object that appeared in the given time interval. To this end, the Cartesian product is used to connect instances of any two consecutive reporting times, and the probability of a resultant sequence is the product of the probabilities of all instances on that sequence. Among all generated sequences, those having two consecutive instances not topologically connected are invalid and should be removed, and those remaining sequences are assigned normalized probabilities eventually. As a result, we consider the flow of each candidate semantic location as the summed probabilities of all valid indoor paths that have passed that semantic location.

The summary of its modeling of contexts and data uncertainties is as follows.

- **Analytical Contexts**. First, a dual-form graph is generated from the original connectivity base graph, in which reference points are regarded as nodes and the passed semantic locations between two reference points are maintained at edges. In this way, it is easy to find relevant semantic locations given any two consecutive instances (reference points). Second, R-trees are used to index both semantic locations and positioning data, which enable efficient spatial joins between semantic locations and positioning instances in the search.
- **Spatial Data Uncertainties**. As mentioned above, positioning uncertainty is modeled using the instance-based representation while low sampling uncertainty is modeled using an interval uncertainty model, i.e., a set of possible instance sequences.

To speed up the search, several supporting techniques are proposed. First, to avoid the explosive instance sequences generated by the Cartesian product, indoor topology based pruning and semantic information based instance grouping are used for data reduction. Second, spatial joins over the semantic location R-tree and the positioning instance R-tree are utilized, prioritizing those semantic locations (and their parent regions) that potentially cover more passing moving objects.

### 4.2. Path planning

Modern buildings are becoming increasingly large and complex, e.g., the Dubai Mall covers an area of over 12 million square feet (equivalent to more than 50 soccer fields)^6^, the King Fahd International Airport is 3.5 million square feet^7^, and the Louvre museum covers a total area of 782,910 square feet of galleries space, as big as 280 tennis courts^8^. It is difficult for people to find an appropriate path in such a large and complex building, especially when they are new visitors. Moreover, to better cater to the user's navigation needs, some important contextual information, such as semantic information of POIs, temporal variation of doors, and dynamic crowds, should be considered in the pathfinding, rather than simply returning the shortest path between the source point and the target point. Correspondingly, we present the following techniques for *keyword-aware path planning* (Feng et al., 2020), *temporal-variation aware path planning* (Liu et al., 2021a), and *crowd-aware path planning* (Liu et al., 2021c).

#### 4.2.1. Keyword-aware path planning

In the previous study (Feng et al., 2020), we focus on finding indoor paths that can cover user-specific keywords (e.g., “cola” and “toilet”). Specifically, an *indoor top-k* *keyword-aware routing query* is defined, which returns the *k* best routes from the start point to the target point that are not longer than a distance constraint and have the highest ranking scores defined as a linear combination of the route's keyword relevance and its length.

According to the workflow defined in the proposed framework, the modeling is as follows.

- **Analytical Contexts**. A connectivity base graph is built to maintain the geometric and topological information used for routing. For semantic information, two types of keywords are considered. An identity word (i-word) is an indoor partition's unique semantic name such as *McDonald's* and *toilet*, while a thematic word (t-word) is the description of an i-word such as *hamburger* and *cola* for *McDonald's*. Two mappings are used to organize i-words and t-words, i.e., finding a group of relevant t-words for a given i-word and finding relevant i-words for a given t-word. Two other mappings are used to maintain the one-to-many relationships between an i-word and a set of associated partitions. The mappings allow for updating in case of changes of semantic information.
- **Spatial Data Uncertainties**. As the analysis does not involve any internal entities and positioning data, no uncertainty is modeled.

The aforementioned context model supports the top-*k* keyword-aware routing query answering in the following aspects. First, the maintained relationships between keywords and partitions are used to compute the keyword relevance score of a given route represented as a sequence of passed partitions. The keyword relevance score is further combined with the route distance in a weighted manner for ranking the most suitable indoor paths for the user query. Second, geometric and topological information of the building space is used to design a set of pruning rules, reducing the search space of candidate paths between the source and the target. Enabled by different pruning rules, two search strategies are proposed. In particular, the topology-oriented expansion strategy expands a partial path to the next graph node^9^, computes the ranking score of the partial path, and prioritizes processing those partial paths with higher ranking scores until *k* complete paths are found. In contrast, the keyword-oriented expansion strategy first finds all keyword-relevant partitions and then searches for complete paths by orchestrating these keyword-relevant partitions and complementing other in-between partitions based on indoor topology.

A follow-up work (Chan et al., 2021) considers more sophisticated contexts in keyword-aware routing, such as the time cost to spend on a POI and the *category word* of a POI. In the modeling, these static contexts of building space are linked to the partitions in the base graph.

#### 4.2.2. Temporal-variation aware path planning

The previous work (Liu et al., 2021a) aims to find the shortest indoor paths for users while considering the temporal variations of doors and blocking of rooms during their navigation. Such a pathfinding problem reflects the real-world scenarios where many doors have regular opening and closing times and many rooms may be temporarily occupied and unavailable for routing use.

To solve the problem, we consider the modeling process as follows.

- **Analytical Contexts**. Static contexts including geometric and topological information are maintained in the base graph, like partitions' shapes and locations of doors. Dynamic contexts mainly consist of doors' *Active Time Intervals* and types of doors/partitions, where the former records each door's opening and closing times and the latter indicates whether a door/partition is private or public. All these dynamic contexts are linked to the graph and allow for updating. To speed up pathfinding, an indoor temporal-variation index is built upon VIP-tree (Shao et al., 2016). The tree structure is constructed by hierarchically gathering those topologically connected partitions such that each internal tree node corresponds to an interconnected area. Moreover, each internal node links to a distance cube that materializes the time-parameterized shortest path information among the doors covered by the internal node.
- **Spatial Data Uncertainties**. Similar to the work above, no spatial data uncertainty is involved.

Based on the constructed context model, synchronous and asynchronous strategies are proposed to deal with temporal variations during the pathfinding over the graph. In particular, synchronous pathfinding checks temporal variations whenever a node expansion is needed whereas asynchronous pathfinding enjoys lazy computations as graph checkpoints are used to indicate the topological changes. An accelerated search using the indoor temporal-variation index is also proposed. This search first finds leaf nodes covering the source and the target, then finds the lowest common ancestor for the two leaf nodes, and finally connects the source to the target *via* their common ancestors with materialized shortest path information.

#### 4.2.3. Crowd-aware path planning

The work (Liu et al., 2021c) aims for finding proper indoor paths by taking into account the crowded people en route. Two different query types are studied. The *indoor crowd-aware fastest path query* (FPQ) returns the path with the shortest traveling time in the presence of the lag caused by crowds, and the *indoor least crowded path query* (LCPQ) finds a path encountering the least number of objects.

To answer these two queries, contexts and data uncertainties are handled as follows.

- **Analytical Contexts**. Static topological and geometric information such as partitions' areas and shapes are maintained in the base graph. The dynamic contexts of internal entities, such as the populations of partitions at each time interval and the flow *via* a door, are modeled by external models that are linked to the base model. They serve to estimate the traveling time and number of encountered objects at a particular time during en route.
- **Spatial Data Uncertainties**. Positioning data is used to model and predict the time-evolving door flows and partition populations, whose uncertainty will however affect the estimation of traveling time and the number of encountered objects. Without deriving the uncertainty region of each individual object, we aggregate the uncertain positioning data to obtain uncertain flows at different time points, and fit it with a Poisson distribution. To deal with the uncertainty at the flow level, we capture the relationship between inflows and outflows of partitions according to the indoor topology, and exploit it to design a recursive flow rectification scheme for accurate population estimation.

The Dijkstra-based search over the graph model along with time-evolving population estimators is used to process FPQ and LCPQ. Two exact and two approximate estimators are provided in the query processing framework. Among the two exact estimators, the global one executes flow rectification over all partitions while the local one focuses on rectifying the inflow/outflow of a relevant partition. The two approximate estimators accelerate the query processing at the cost of accuracy. One skips the outflow rectification of those dependent partitions when deriving the population of a relevant partition, while the other optionally uses probability functions to predict populations instead of the timestamp-by-timestamp derivation of populations.

### 4.3. Semantic trajectory generation

Translating raw indoor positioning sequences into human-readable, concise indoor semantic trajectories is practically useful. Various downstream applications, such as querying, visualization, and recommendation, can all benefit from such semantics-oriented representation. Following many existing studies on outdoor semantic trajectories (Parent et al., 2013; Yan et al., 2013), an object's indoor semantic trajectory is captured as a sequence of triples in the form of (*s, e*, τ), meaning that the object has a mobility event *e* ∈ {stay, pass-by} within a semantic region *s* during the time interval τ. Semantic trajectory generation is a complex process. A layered framework for constructing indoor semantic trajectories has been introduced in previous studies (Li et al., 2018a, 2020b), encompassing the subtasks of data cleaning, semantic annotation, and sequence completion. A follow-up work (Li et al., 2020a) focuses on improving the performance of semantic annotation. In these studies, sufficient consideration of contexts and data uncertainty is a prerequisite for achieving decent performance in generating indoor semantic trajectories.

Following the proposed framework, the modeling process is executed as follows.

- **Analytical Contexts**. On top of the connectivity base graph, the information of those semantic regions is maintained by hashtables where each semantic region is mapped to a set of indoor partitions. The topological relationships between semantic regions are also derived and added to the base graph. Indoor partitions are indexed by R-trees such that raw positioning records can be efficiently mapped to the covering indoor partition and further mapped to the covering semantic region.
- **Spatial Data Uncertainties**. To facilitate the cleaning of historical positioning data, an interval uncertainty model is employed, capturing the possible object whereabouts as the space-constrained intersection of two circles (Li et al., 2020b). Subsequently, to complete the missing triples in a generated semantic trajectory, the possible movement across the unobserved time interval is modeled as a set of possible paths on the connectivity base graph. The historical data is used to calculate the prior transition probabilities between two semantic regions such that the missing movement between semantic regions can be modeled and inferred using probabilistic approaches.

Finally, we briefly describe how these models for contexts and data uncertainties support the subtasks of semantic trajectory generation:

1. For *positioning data cleaning*, the interval uncertainty model is used to identify outliers in the raw data. Particularly, any observed location falling out of the derived uncertainty region is determined as an outlier, and a sample drawn from the uncertainty region will substitute the outlier.
2. For *semantic annotation*, positioning sequence is segmented using a density-based clustering approach. In particular, a segment with low spatiotemporal density is likely to match a pass-by event; otherwise, high spatiotemporal density indicates a stay event. Once events have been determined, the corresponding semantic region is estimated as the one occupying the largest fraction of the segment's combined uncertainty region. This method determines the event and semantic region sequentially, which may bring about chained errors. Therefore, probabilistic graphical models (Li et al., 2020a) have been exploited to jointly model the matching probabilities of semantic regions and those of events as well as learn their dependencies along the time. To model the energy functions in such a graphical model, indoor geometric information, and semantic information are exploited.
3. For *sequence completion*, the missing triples corresponding to the low sampling issue of the raw data are inferred. Specifically, the historical movement patterns among semantic regions are modeled as transition probabilities between graph nodes. Subsequently, the most likely path between two generated triples is found as the one with maximum posterior probability.

### 4.4. Distance monitoring

The COVID-19 pandemic has endangered people's life since the beginning of 2020. As of August 22, 2022, it had caused more than 6 millions deaths^10^. One of the effective approaches to contain the spread of the virus is to keep proper social distancing (e.g., at least 1 m apart) between people. This is particularly important in buildings like high-risk workplaces (e.g., people in nursing homes and quarantine hotels). In the following, we introduce *continuous social distance monitoring* (SDM) (Chan et al., 2022), which aims to monitor and predict the pairwise distances between moving objects (people) in real-time. In particular, given a set of moving objects in an indoor space, SDM identifies all object pairs that are going to form close contact, i.e., having a distance smaller than a pre-defined threshold (e.g., 1 m) within a near future (e.g., in 5 s or so). One example application is that a museum or gallery can integrate this social distance monitoring into their guide app, which allows visitors to keep track of their distances from others while visiting the exhibition. It can suggest further actions to those contact visitors by alerting them.

In this task, the modeling process is as follows.

- **Analytical Contexts**. To maintain the static context including the geometric and topological information of the building, a connectivity base graph is used. Door-to-door shortest distances and partition-to-partition shortest distances are pre-computed and stored in two matrices. The internal entities of interest are the indoor moving objects (people), and they are maintained in the buckets linked to the covering partition. When a new location update is received, the bucket information of the corresponding object will be updated.
- **Spatial Data Uncertainties**. Both positioning inaccuracy and low sampling issues are considered in modeling object positions. To deal with uncertainties caused by the positioning inaccuracy, we use the circle-based representation combined with different distance decaying functions. However, in the implementation, we draw instances from the circle-based representation as such instances fit in arbitrary distributions and make computations easier. To deal with uncertainties caused by low sampling issues, the interval uncertainty region model is used, corresponding to Case 4 (circle-based representation) in Equation (1).

To process SDM efficiently, a set of acceleration techniques are proposed. First, to reduce the number of pairwise distance computations, we prune unpromising object pairs based on the floors they are located on, the indoor topology, and their probability distributions. Second, we propose a batch processing strategy to group nearby objects into one large group, and update the distances between the objects within and outside the group in one pass.

## 5. Future directions

Above, we have introduced recent advances in addressing complicated analytical contexts and spatial data uncertainties in SDAIB. Next, we discuss new opportunities that emerging technologies bring to SDAIB.

- **Decentralized and edge-resident SDAIB**. In the past decade, SDAIB tasks have been mainly run in a centralized manner, which potentially incurs high costs in the data center for data integration, computation, and storage. Currently, edge computing (Mao et al., 2017) emerges as a novel paradigm, pushing the data processing as close as possible to the place where the data is generated. In this way, reduced computation workload of the data center and improved service responsiveness are achieved. There may be hundreds or thousands of IoT devices in an intelligent building environment, using different working mechanisms to collect, transmit, and process data. Spatial data analyses feature inherent locality, i.e., data generated locally can be analyzed locally. This means that the main process of a spatial data analysis task is very suitable for running in decentralized devices resident in the IoT edge. However, it is nontrivial to realize such a decentralized, edge-resident SDAIB. Specifically, IoT devices in intelligent buildings are heterogeneous and dynamic, with distinct computing capabilities and life cycles. To make appropriate task decomposition and task assignment among IoT devices, new techniques for profiling IoT devices, modeling tasks' objectives (e.g., processing latency, accuracy, and transmitted data volume), and efficient coordination are required. Recent efforts (Ma et al., 2019; Li et al., 2022) have been made to manage and analyze spatial and spatiotemporal data in the IoT edge computing environments.
- **Privacy-preserving SDAIB**. In the new technology ecosystem, another important issue is data security and privacy protection. Compared with data centers, IoT devices are less protected, and their data computation and storage face security risks. In the foreseeable future, more and more spatial data collected in intelligent buildings will be anonymized, obfuscated, encrypted, or removed, which will bring greater challenges to analyses. On the one hand, the efficiency of analysis will potentially be degraded due to more complex data security schemes; on the other hand, privacy-enhanced data may lead to information loss and thus degradation of the results' effectiveness. Some potential techniques can be applied to privacy-preserving SDAIB. First, representation learning techniques (Kumar et al., 2021) can be introduced into a hierarchical analysis process from end devices to IoT devices to the data center, in which generated data encodings are exchanged in the IoT and the network, and end devices and the data center take the normal input and output. Second, data summaries (Siddique and Eldawy, 2018), specific to particular data analysis tasks, can be used in scenarios that are fault-tolerant. Finally, for some model-driven analyses, federated learning (Kumar et al., 2021) and machine learning in Blockchain (Chen et al., 2021) can help avoid sharing sensitive data while maintaining decent performance.
- **Lightweight and energy-saving SDAIB**. Last but not least, the realization of lightweight and energy-efficient data analysis is of great benefit to intelligent buildings. IoT-enhanced buildings require a lot of energy to acquire data and interact with people and the environment. Hence, it is necessary to cut ineffective operations from data analysis, for sustainable intelligent building applications. We illustrate potentially applicable technologies from several perspectives. (1) From a data perspective, how to sample, retain, and discard data is worth investigating. Many sensors collect redundant data at high frequency, therefore, data sampling strategies based on reinforcement learning (Yoon et al., 2020) will be highly usable, e.g., to adaptively change the sampling rate or the buffer size according to the objective of the analysis task. (2) From a computational perspective, identifying and removing invalid or inefficient operators in the analysis can be explored. For some machine learning tasks, lightweight techniques (Menghani, 2021) such as model compression, knowledge distillation, and parameter pruning have been applied in computer vision and natural language processing fields, but how to design light models for spatial data and spatiotemporal data in IoT scenarios still remains open.

Based on the above, we envision an end-edge-cloud architecture for SDAIB, which encompasses various data privacy protection schemes and decentralized algorithms built on them. This architecture also integrates a coordinator module, which provides a quality-aware and energy-efficient mechanism to decompose tasks and assign them to heterogeneous and dynamic IoT devices.

To support the aforementioned future directions, the unified modeling framework proposed in Section 3 should be further improved. In terms of *context modeling*, the framework should be able to represent more complex and diverse IoT devices and building environments. On the one hand, compared to current manually designed models, automated means such as semantic parsing and extraction (Teng et al., 2018; Guo et al., 2021) can be developed to extract contexts from the physical world; on the other hand, data structures with more powerful expression capabilities such as heterogeneous graphs and hypergraphs (Zhou et al., 2006) can be introduced to the modeling framework. In terms of *data uncertainty modeling*, the framework should support the decentralized data setting (Li et al., 2022), i.e., heterogeneous computing nodes generate and consume data with different mechanisms. In this case, the framework should be able to model and analyze the spatial data uncertainty of heterogeneous data nodes, and the merging of such spatial data in different degrees or types of uncertainty.

## 6. Conclusion

In this article, we focus on advanced techniques of spatial data analysis for intelligent buildings (SDAIB), and identify two general technical challenges in SDAIB, namely the complicated analytical context and inherent spatial data uncertainty. We then revisit recent advances in modeling analytical context and spatial data uncertainty. By summarizing the technical highlights of these modeling approaches, we propose a unified modeling framework that creates a roadmap for handling various analytical contexts and spatial data uncertainties. We demonstrate the support of this unified modeling framework for several SDAIB tasks. Finally, we discuss the future directions of SDAIB in the presence of emerging technologies, which may inspire researchers and practitioners in constructing innovative intelligent building applications.

## Data availability statement

The original contributions presented in the study are included in the article/supplementary material, further inquiries can be directed to the corresponding author/s.

## Author contributions

HLi and HLu contributed to the conception and design of this work. All authors contributed to the article and approved the submitted version.

## Funding

The authors' work was supported by the Independent Research Fund Denmark (Grant No. 8022-00366B) and the EU MSCA program (Grant No. 882232).

## Conflict of interest

The authors declare that the research was conducted in the absence of any commercial or financial relationships that could be construed as a potential conflict of interest.

## Publisher's note

All claims expressed in this article are solely those of the authors and do not necessarily represent those of their affiliated organizations, or those of the publisher, the editors and the reviewers. Any product that may be evaluated in this article, or claim that may be made by its manufacturer, is not guaranteed or endorsed by the publisher.

## Footnotes

^1^ https://www.researchandmarkets.com/reports/5235835/global-smart-buildings-market-by-component

^2^Positioning reference points are the predefined locations in the building, providing calibration information and ground truth for positioning algorithms (Liu et al., 2007).

^3^The maximum speed can be maintained object-wise, as long as prior knowledge is available.

^4^Different notations are used to refer to the base indoor graph structure with partitions as nodes and doors as edges (Jensen et al., 2009a; Worboys, 2011; Afyouni et al., 2012; Lu et al., 2012). In this study, we use the connectivity base graph following an early work (Jensen et al., 2009a).

^5^ https://en.wikipedia.org/wiki/New_Town_Plaza

^6^ https://en.wikipedia.org/wiki/The_Dubai_Mall

^7^ https://en.wikipedia.org/wiki/King_Fahd_International_Airport

^8^ https://en.wikipedia.org/wiki/Louvre

^9^A partial path, in contrast to a complete path, represents those paths that start from the source but have not reached the target.

^10^ https://covid19.who.int
